# Psychosocial risk factors of myocardial infarction and adverse effects of streptokinase in public sector hospitals

**DOI:** 10.12669/pjms.314.6803

**Published:** 2015

**Authors:** Saira Afzal, Muhammad Arif Khan, Hafiz Muhammad, Ayesha Ashraf, Maria Afzal

**Affiliations:** 1 Saira Afzal, MBBS, MCPS, M.PHIL, FCPS Chairperson and Head, Department of Community Medicine, King Edward Medical University, Lahore, Pakistan; 2 Muhammad Arif Khan, MBBS, M.SC APMO, Department of Community Medicine, King Edward Medical University, Lahore, Pakistan; 3 Hafiz Muhammad Waqas khan, MBBS Department of Medicine, Mayo Hospital, Lahore, Pakistan; 4 Ayesha Ashraf, MBBS Department of Medicine, Mayo Hospital, Lahore, Pakistan; 5 Maria Afzal, M.SC Department of Psychology, Government College University, Lahore, Pakistan

**Keywords:** Streptokinase administration, Myocardial infarction, Morbidity and Mortality

## Abstract

**Objectives:**

This study investigated the psychosocial risk factors of myocardial infarction and time related adverse effects of administration of streptokinase on short-term morbidity and mortality in patients with ST-segment-elevation myocardial infarction (STEMI).

**Methods:**

One hundred patients with STEMI treated with streptokinase in the hospital setting were prospectively enrolled in the study. The primary outcome parameter was the incidence of major adverse cardiac events. During hospital stay the psychosocial and demographic risk factors were also investigated.

**Results:**

The overall mortality rate was similar in both groups and it was not significant. (5.7% vs 14.5%; *P* = 0.18). The number of recurrent chest pain was significantly higher in the group 2 compared to the group 1 (25% vs 62.5%; *P* = .01). The number of hypotesion was significantly higher in the group 1 as compared to the group 2 (30.7% vs 6.2%; *P* = .009). The demographic and psychosocial risk factors were recorded.

**Conclusions:**

The early intravenous administration of streptokinase in the hospital setting leads to a reduced rate of major cardiovascular events compared to delayed administration beyond 2 hours. However, mortality rates were not significantly affected. Secondary prevention should be targeted on modifiable demographic, dietary, and psychosocial risk factors of STEMI.

## INTRODUCTION

Streptokinase (SK) is a bacterial product secreted by Streptococci. It is protein and as it is bacterial product so human body has the ability to develop immunity against it.^1^ It was developed as a thrombolytic drug that was used earlier for haemothorax, pleural exudates, tuberculous meningitis treatment and then used for Myocardial infarction, pulmonary embolism and empyema. It had been used in developing nations due to its low cost, and affordability whereas most of the developed nations are currently using (t-PA) tissue plasminogen activator. The side effects of streptokinase include pyrogenic reactions like malaise, headache, arthralgia and occasionally nausea and febrile responses. It was also known as wonder drug.^2^ Many trials had shown that it had reduced mortality in MI patients when administered within 3-4 hours of onset of symptoms like chest pain. It could be administered both intravenously and intra coronary. Its over dosage should be treated promptly, otherwise it could cause fatal bleeding. The secondary prevention in myocardial infarction had its definite role in improving survival of the patients and targeting risk factors of myocardial infarction.^3^ ST elevation myocardial infarction (STEMI) is defined as ST elevation on ECG, that is the electrical manifestations of patho-physiological changes after thrombotic occlusion of an epicardial coronary artery.^4^

It had been acknowledged through a cohort study that in acute myocardial infarction, the time between onset of pain and beginning of thrombolytic therapy with Streptokinase strongly influences patency rates by restoring the blood flow to the ischemic myocardium.^5^ Patients treated with streptokinase were compared to untreated patients. Patients with streptokinase therapy had lower need for nicomorphine and had less severe shorter duration pain. SK therapy when initiated within first 6 hours of acute myocardial infarction reduced mortality and the therapy was most beneficial for those patients with anterior myocardial infarction and those who could receive therapy within the first 2-3 hours from the onset of symptoms.^6^,^7^

A study carried out to obtain a preliminary data on the relative clinical utility of direct coronary angioplasty compared with that of intravenous streptokinase for myocardial infarction patients showed that SK administration could be preferred over coronary angioplasty for most patients because of shorter time to the treatment.^8^ It had also been shown that streptokinase administered less than one hour after the onset of symptoms of acute myocardial infarction followed by angioplasty of the infarct artery resulted in the preservation of the left ventricular function where as therapy given after two hours had only a limited effect in patients.^9^

The psychosocial risk factors associated with cardiac diseases are anxiety, depression, work overload, stress, sleep disturbance and socioeconomic status in both sexes and at all ages in different regions around the globe.^10^ The findings suggested that secondary prevention should be based on those principles worldwide and should not ignore the social and cultural differences among different geographical regions. However the strategies and health education to prevent cases of myocardial infarction should only be affective if considering the importance of psychosocial risk factors in that region leading to AMI.^11^

Patients with acute myocardial infarction are at high risk of mortality within the first hours after onset of myocardial ischemia. Therefore, intervention should be started as soon as possible. This study investigated the psychosocial risk factors of myocardial infarction and adverse effects of the hospital administration of streptokinase on short-term morbidity and mortality in patients with ST-segment-elevation myocardial infarction (STEMI).

## METHODS

The quasi experimental research was conducted in Medicine and Cardiology wards of Public sector hospitals in Lahore in order to investigate the psychosocial risk factors of Myocardial infarction and side effects of Streptokinase administration in post myocardial infarction patients. The selected public sector hospitals were situated in the centre of Lahore, easily approachable by the community, very busy emergency wards, most of the medicine were available free of cost to the patients, and patients could be shifted from their houses to the hospital emergencies by Ambulance Service 1122 provided by government.

Study duration was from August 2012 to July 2014. Study subjects were willing to participate in the research study and their information was kept confidential. One hundred patients aged 30-80 years with STEMI in the hospital setting were prospectively enrolled. Streptokinase was given according to the guidelines.^2^ Exclusion criteria is presented in the Table-I. Convenient sampling technique was used. Written informed consent was obtained from all selected study subjects and there was no conflict of interests. No funding or support was received for this study. The group1 consisted of patients who had intravenous administration of streptokinase within 2 hours after prolonged chest pain and group2 included patients who received streptokinase beyond 2 hours but within 6 hours after the primary event.

**Table-I:** Check list for exclusion criteria of streptokinase administration.

S. N	Exclusion criteria
1	Blood Pressure (mm Hg).
	1. Sustained B.P>180/110
	2. Hypotension B.P<90
2	Past history.
	1. Intracranial hemorrhage
	2. Stroke within one year
	3. Past Q wave MI
	4. Valvular heart diseases.
3	Known neoplasm
4	Known Acute pancreatitis
5	Active internal bleeding
6	Menstruation/Vaginal bleeding
7	Patients on anticoagulation therapy
8	Known bleeding diathesis
9	Active peptic ulcer and Gastrointestinal bleeding in previous one month.
10	Suspected Aortic Dissection
11	Recent Major Surgery in previous 3 weeks.
12	Pregnancy
13	Streptokinase related thrombolysis in 5 days to 2 years.
14	Hypersensitivity to streptokinase
15	Late presentation of MI after 6 hours of chest pain

Eligible patients received 1.5 million units of streptokinase intravenously in normal saline over one hour. Patients were meticulously monitored during streptokinase administration and regularly for 90 days after primary event. Blood samples were investigated for hematological and biochemical investigations. Echocardiography was used to see pericarditis and complications.

The time of streptokinase administration was measured in hours after onset of prolonged >30 minute chest discomfort or chest pain. The post myocardial complications included recurrent chest pain defined as >30 minutes pain after streptokinase administration, pericarditis defined as hypoechoic shadows on echocardiography, hypotension defined as systolic B.P less than 90 mmHg.

The laboratory investigations recorded were blood sugar levels, blood cholesterol levels and raised cardiac enzymes in patients with ST elevations on electrocardiogram (STEMI). All the changes were serially monitored and follow up was maintained on pretested structured checklists and questionnaire designed for the research. When the patients of Myocardial infarction became stable and responsive then they were interviewed for identification of psychosocial risk factors leading to Myocardial infarction. The operational definitions of psychosocial variables in the study were as follows: Depression is a state of low mood and aversion to activity that can affect a person’s thought, behavior, feelings and physical well being. Generalized Anxiety Disorder is a psychological and physiological state characterized by somatic, emotional, cognitive and behavioral components. ICD 10 classification was used to diagnose depression, generalized anxiety disorder (GAD) and psychological stress by psychiatrist. Person was literate who could read and write paragraph (3 lines) in national/regional language with comprehension. Smokers were those who smoked cigarettes, shisha (water-pipe) and/or cigars at least once a day were defined as smokers. Sedentary lifestyle was defined as sitting position in office/house ≥ 9 hours and performing less than 25-30 minutes of physical activity per day. Lack of Sleep was defined as sleep less than 8 hours in 24 hours for past two consecutive months. Poverty was monthly income less than 10,000 rupees from all the sources.

The indicators of successful thrombolysis were measured as disappearance of chest pain and resolution of ST segment elevation by two thirds at 90 minutes post thrombolysis with or without reperfusion arrhythmias. Patients of STEMI who had reperfusion failure were referred for rescue Percutaneous Coronary Intervention (PCI). Both groups who received streptokinase also received low molecular weight (LMW) heparin (Enoxaprin), aspirin, Angiotensin Converting Enzyme inhibitors, beta blockers and statins. Previous medical records and past histories wherever available were thoroughly reviewed for various risk factors. Upper gastrointestinal endoscopy was done where gastrointestinal bleed was suspected on vomitus or stools. The endpoint of this research was documentation of side effects including composite of recurrent chest pain, pericarditis, hypotension or systolic B.P<90 mm Hg, arrhythmias, bleeding, anaphylactic reactions, or mortality within 90 days of hospital stay.

## RESULTS

Among MI cases (n=100),*52% (mean 0.52± 0.502SD) came within 2 hours after chest pain and 48% within 6 hour after chest pain. In group 1 SK was administered in 52% patients within 2 hours. In group2, 48% cases received SK beyond 2 hours but within 6 hours. The chest pain was relieved in 87% (mean 0.87± 0.33) patients in 6 to 12 hours*.

During monitoring after SK administration 7.6% patients of MI in group1 vs 6.2% patients in group2 suffered from arrhythmias (7.6% vs 6.2%; Chi-square=0.07, p=0.79), 13.4% cases suffered from bleeding in group1 and 10.4% in group2 (13.4% vs 10.4%; Chi-square=0.17, P = .67), bleeding was observed from gastrointestinal tract, macroscopic hematuria, venepuncture site and skin.

About 11.5% cases had pericarditis in group1 vs 37.5% in group2 on echocardiography (11.5% vs 37.5%; Chi-square=5.67, P = .017), and hypotension was recorded in 30.7% in group1 vs 6.2% in group2 (30.7% vs 6.2%; Chi-square=6.76, P = .009). Moreover, anaphylactic reaction in 3.8% in group1 and 4.1% in group2 (3.8% vs 4.1%; Chi-square=0.01 P = .9), recurrent chest pain in 25% in group1 vs 62.5% in group2 (25% vs 62.5%; Chi-square=5.75, P = .016), and fever in 30.7% in group1 and 29.1% in group2 (30.7% vs 29.1%; Chi-square=0.02, P = .89) were documented. (Table-II)

**Table-II:** Comparison between group 1 and group 2.

Variable	Group1 (n=52)	Group2 (n=48)	Chi square value	p value
Mortality	3 (5.7%)	7 (14.5%)	1.76	0.18
Recurrent chest pain	13 (25%)	30 (62.5%)	5.75	0.016
Pericarditis	6 (11.5%)	18 (37.5%)	5.67	0.017
Hypotension	16 (30.7%)	3 (6.2%)	6.76	0.009
Arrythmias	4 (7.6%)	3 (6.2%)	0.07	0.79
Bleeding	7(13.4%)	5 (10.4%)	0.17	0.67
1. GIT bleed			
2. hematuria			
3. Others			
Anaphylactic reaction	2 (3.8%)	2 (4.1%)	0.01	0.9
Fever	16 (30.7%)	14 (29.1%)	0.02	0.89

The risk factors found were illiteracy (31%), dyslipidaemia (78%), depression (28%), generalized anxiety disorder (36%), psychological stress (87%), smoking (66%), peripheral vascular disease (1%), sedentary life style (51%), poverty (46%) and lack of sleep (69%). Door to needle time was found in 2 hours or less (52%) and after 2 hours but with in 6 hour (48%) after primary event. (Table-III)

**Table-III:** Demographic, medical and psychosocial variables.

Variables	Frequencies	Percentages
*Age of patient*		
a-30 or above but below 80	a-75	a-75.0
b-80 and above	b-25	b-25.0
*Gender of patient*		
a-male	a-81	a-81.0
b-female	b-19	b-19.0
*Education of patient*		
a-literate	a-69	a-69.0
b-illiterate	b-31	b-31.0
*Religion of patient*		
a-Muslim	a-98	a-98.0
b-non-Muslim	b-2	b-2.0
*Dyslipidaemia*		
a-Yes	a-78	a-78.0
b-No	b-22	b-22.0
*Diagnosed Peripheral Vascular disease*		
a-Absent	a-99	a-99.0
b-Present	b-1	b-1.0
*Door to needle time*		
a-within 6 hour after chest pain	a-48	a-48.0
b-within 2 hours after chest pain	b-52	b-52.0
*Psychological stress*		
a-yes	a-87	a-87.0
b-no	b-13	b-13.0
*Smoking/Tobacco use*		
a-yes	a-66	a-66.0
b-no	b-34	b-34.0
*Generalized Anxiety disorder*		
a-yes	a-36	a-36.0
b-no	b-64	b-64.0
*Depression*		
a-yes	a-28	a-28.0
b-no	b-72	b-72.0
*Sedentary life style*		
a-yes	a-51	a-51.0
b-no	b-49	b-49.0
*Sleep less than 8 hours*		
a-yes	a-69	a-69.0
b-no	b-31	b-31.0
*Poverty*		
a-<10000Rs	a-46	a-46.0
b->10000 or equal	b-54	b-54.0

## DISCUSSION

Streptokinase is secreted by streptococci.^1^ It was the first thrombolytic drug that was used for myocardial infarction. Although more than 80% of the global burden of cardiovascular disease is contributed by low-income and middle-income countries, research and evidence of the importance of risk factors is largely derived from developed countries.^10^ Therefore, the effect of such factors on risk of Myocardial infarction in most regions of the world is unknown. Moreover time related effects of streptokinase required investigations.

In this study Streptokinase was administered in 52% patients within 2 hours and 48% cases within 6 hours after chest discomfort or chest pain which relieved symptoms in 87% of patients (mean 0.87±0.338SD) while in another study conducted in US showed that 30 patients presented to the hospitals in a mean time of 1.21±1.08 hours and treatment commenced in a mean time of 2.77±1.3 hours after the onset of symptoms, 86.7% patients were reperfused initially and 2 were found re-occluded within first 48 hours.^12^ The study conducted on myocardial ischemia patients, showed re-occlusion in 20% of patients.^13^

The study 3.8% in group1 vs 4.1% in group2 were diagnosed as cases of anaphylactic reactions after SK administration because it is a bacterial product and act as a foreign body. The similar results were found in research study in USA where cases of IgE mediated cases of anaphylactic reactions were observed.^14^

In this research patients were followed and 11.5% in group1 and 37.5% in group2 developed post myocardial pericarditis, while similar results were observed in study conducted in New York. It was reported that pericarditis was developed in 3 of 38 patients (8%) at day 0, in 2 of 44(5%) at day 1, in 8 of 43(19%) at day 3.^15^

Mortality rate in this study was 5.7% in group1 vs 14.5% in group2, while in another study conducted in USA, mortality rate was 3.4% recorded. It was also seen that only one mortality occurred after 2 months of hospital stay and 51 out of 55 patients of MI in USA were back to work after treatment.^16^

According to current study anxiety was a risk factor for STEMI and myocardial infarction. Research studies conducted in the past found that anxiety was a major risk factor contributing to myocardial infarction.^17^

In present study lack of sleep that was less than 8 hours in 24 hours in past 2 months was found in 69% patients of myocardial infarction. Another research study conducted also showed that lack of sleep was a risk factor for ischemic heart disease.^18^

According to the studies conducted in the past poverty, sedentary life style, stress and depression were the risk factors of Ischemic Heart diseases. It was also found that strong association of smoking was present for IHD.^19^ Avoiding sedentary life style by performing regular exercise for at least 30 minutes for five days a week reduced the risk of myocardial infarction.^20^

Smoking was found risk factor in 36% and obesity in 20% of Myocardial infarction or STEMI.^19^ Lack of exercise was the risk factor in 7-12% of cases of Myocardial infarction.^20^ Other risk factors included psychological stress such as job stress in 3% of cases, and chronic high psychological stress levels.^21^

In other research studies tobacco smoking, including secondhand smoke, Lack of physical activity, psychosocial factors, low socioeconomic status or poverty, social isolation and negative emotions were found to increase the risk of Myocardial infarction and its complications. Socioeconomic factors such as an illiteracy and lower income were correlated with a higher risk of Myocardial infarction.^22^ Similar risk factors were found in present study.

In a study involving 102 patients of Acute Myocardial infarction who received streptokinase 1.5 million units within 12 hours of onset of chest pain had successful reperfusion in 47% patients. The adverse effects reported were bleeding, hypotension and allergic reactions. In our study similar adverse effects were found however streptokinase was administered within 6 hours after chest pain and relieve of symptoms were found in 87% patients.^23^

In another study the researchers concluded that shock, recurrent chest pain and ischemia occurred more often in patients of STEMI, which supported our research findings that recurrent chest pain was found statistically significant and more frequency in late administration of streptokinase than early administration.^24^

### Limitations of the study.

There was no control group so that no comparison of adverse effects and risk factors in quantitative data and qualitative data could be made. Government had provided streptokinase free of cost to most of the patients in public sector hospitals thus streptokinase was the preferred therapy for thrombolysis in STEMI. Very few patients were administered other thrombolytic agents (e.g. Alteplase) and accepted procedures in public sector hospitals due to high cost and high financial burden. Thus comparison between different thrombolytic agents could not be made. The quantitative scale like Thrombolysis in Myocardial Infarction (TIMI) grading system that was used to find the flow in the culprit artery as grade3 flow being the goal of thrombolytic therapy was not possible due to limited availability and non affordability of patients. Thus present research showed that reduced complications were significantly associated with timely administration of SK (within 2 hours).

## CONCLUSION

Early intravenous administration of streptokinase in the hospital setting reduced the rate of major cardiovascular events compared to delayed administration beyond 2 hours. Secondary prevention should be targeted on modifiable demographic, and psychosocial risk factors of STEMI including psychological stress (87%), lack of sleep (69%), smoking (66%), sedentary life style (51%), poverty (46%) and illiteracy (31%), depression (28%), and generalized anxiety disorder (36%) in the study population. The targeted approach to the psychosocial risk factors through health awareness can help in reducing disease burden in future.
